# Recent Progress in One- and Two-Dimensional Nanomaterial-Based Electro-Responsive Membranes: Versatile and Smart Applications from Fouling Mitigation to Tuning Mass Transport

**DOI:** 10.3390/membranes11010005

**Published:** 2020-12-22

**Authors:** Abayomi Babatunde Alayande, Kunli Goh, Moon Son, Chang-Min Kim, Kyu-Jung Chae, Yesol Kang, Jaewon Jang, In S. Kim, Euntae Yang

**Affiliations:** 1 School of Civil, Environmental and Architectural Engineering, Korea University, Seoul 02841, Korea; abayomi@korea.ac.kr; 2 Singapore Membrane Technology Centre, Nanyang Environment and Water Research Institute, Nanyang Technological University, Singapore 637141, Singapore; gohkunli@ntu.edu.sg; 3 School of Urban and Environmental Engineering, Ulsan National Institute of Science and Technology, UNIST-gil 50, Ulsan 44919, Korea; moonson619@gmail.com; 4 Graduate School of Water Resources, Sungkyunkwan University (SKKU), Gyeonggi-do 2066, Korea; cmkim1985@gmail.com; 5 Department of Environmental Engineering, Korea Maritime and Ocean University, Busan 49112, Korea; ckjdream@kmou.ac.kr; 6 Interdisciplinary Major of Ocean Renewable Energy Engineering, Korea Maritime and Ocean University, Busan 49112, Korea; 7 Global Desalination Research Center (GDRC), School of Earth Sciences and Environmental Engineering, Gwangju Institute of Science and Technology (GIST), Gwangju 61005, Korea; yesol7964@gist.ac.kr (Y.K.); jwjang@gist.ac.kr (J.J.); iskim@gist.ac.kr (I.S.K.); 8 Department of Marine Environmental Engineering, Gyeongsang National University, Gyeongsangnam-do 53064, Korea

**Keywords:** membrane, electro-conductive, nanomaterial, water treatment

## Abstract

Membrane technologies are playing an ever-important role in the field of water treatment since water reuse and desalination were put in place as alternative water resources to alleviate the global water crisis. Recently, membranes are becoming more versatile and powerful with upgraded electroconductive capabilities, owing to the development of novel materials (e.g., carbon nanotubes and graphene) with dual properties for assembling into membranes and exerting electrochemical activities. Novel nanomaterial-based electrically responsive membranes have been employed with promising results for mitigating membrane fouling, enhancing membrane separation performance and self-cleaning ability, controlling membrane wettability, etc. In this article, recent progress in novel-nanomaterial-based electrically responsive membranes for application in the field of water purification are provided. Thereafter, several critical drawbacks and future outlooks are discussed.

## 1. Introduction

Wastewater reuse and seawater desalination are key strategies for addressing global water shortage [1]. Reliable and cost-competitive water purification technologies are essential in ensuring high water quality and safe drinking water for all. In recent decades, several technologies were developed and successfully applied to wastewater reuse and seawater desalination [1,2,3]. In particular, membrane-based technologies positioned themselves at the center of many mainstream water applications, owing to the enhanced quality of product water, highly efficient and precise removal of pollutants at a relatively low cost and small footprint, and robust operations [3,4,5].

However, despite the wide use of membrane-based technologies, there remain several issues that need to be improved, such as membrane fouling, energy consumption, and low removal efficacy toward some contaminants [6]. Since these issues are mainly associated with the properties of membranes, many studies were focused on the enhancement of membrane characteristics (surface chemistry and pore structure) and the development of advanced membranes using novel building blocks (e.g., aquaporin, one-dimensional (1D) and two-dimensional (2D) nanomaterials) with excellent properties, e.g., antibacterial effect, ultrafast water transport route, and precise molecular sieving [7,8,9].

The development of novel membrane materials by using 1D and 2D materials received much attention over the years [10]. One-dimensional nanomaterials are materials with a singular structure with dimensions outside of the nanoscale and they are represented by nanorods, nanotubes, and nanowires. Two-dimensional nanomaterials, on the other hand, are nanomaterials that extend in a two-dimensional plane with a thickness ranging from a few to tens of nanometers, usually outside the nanoscale (e.g., nanosheets, nanoplates, and nanoflakes) [11]. The dimensionality of 1D and 2D nanomaterials make them possess good electrochemical properties due to their high specific surface area, oriented electronic and ionic pathways, and low electrical resistivity, and their assemblable properties [12]. One-dimensional nanomaterials could be blended with polymer for membrane synthesis or individually vertically aligned so that nanotubes could be used for water transport. The atomic scale smoothness of nanotube walls enables ultra-fast water transport along the nanotubes [13]. However, vertically aligning nanotubes to form a free-standing membrane is challenging due to the technicality of vertical alignment at high density and on a large scale. Therefore, practical applications of 1D nanomaterials have been limited to blending these materials into polymeric membranes. Two-dimensional nanomaterials; however, have been extensively used as membrane material for separation application because of their atomic scale thickness, high mechanical strength and chemical inertness [14]. Two-dimensional nanosheets could be made into a separation membrane by creating a nanopore uniformly on the nanosheet or by stacking 2D nanomaterials into laminar membranes. Thus, the ability of 1D and 2D nanomaterials to be used as fillers during polymeric membrane fabrication or solely arranged to fabricate a free-standing membrane makes them an alternative candidate for membrane used in membrane-based separation process.

One- and two-dimensional nanomaterials such as carbon nanotubes (CNTs), graphenes, and MXenes do not only possess the above-mentioned properties, but also the catalytic ability and high electrical conductivity that can be leveraged for membrane application [8,9], by endowing membranes with electrically conductive and electrochemically catalytic capabilities [8,15,16]. This smarter and versatile membrane platform possessing these electrical and electrochemical functions is referred to as electrically responsive membranes (ERMs). With the assistance of external power, ERMs can conduct additional capabilities that conventional membranes cannot perform. In previous studies, it was demonstrated that electrically responsive membranes (ERMs) can be used to mitigate membrane fouling [17,18,19], monitor membrane fouling propensity [20], self-cleaning [21,22], electrochemically oxidizing organic contaminants [23], controlling permeability and selectivity [24,25,26], energy recovery [27], and controlling membrane wettability [28].

**Figure 1:**
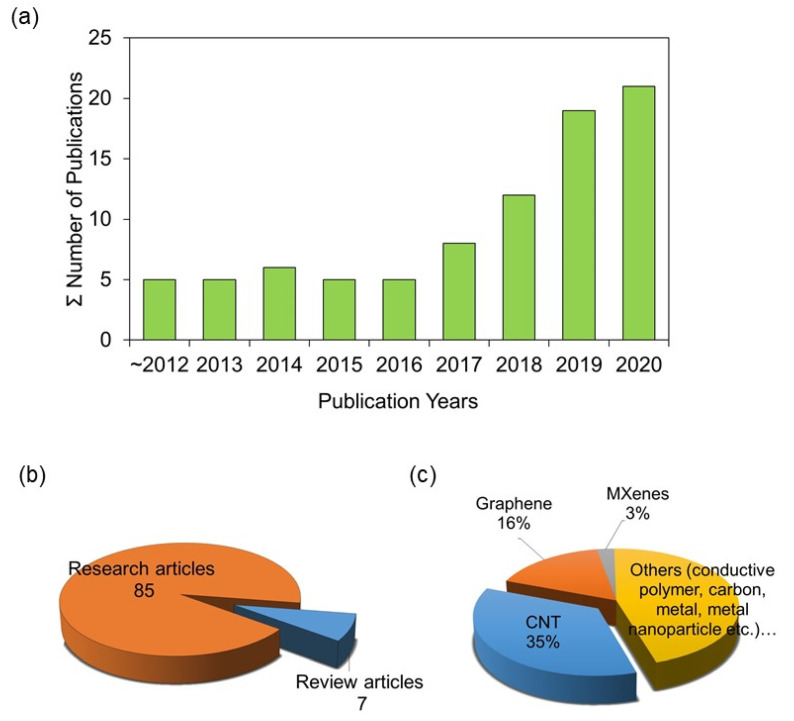
The research trend of the published papers related to electrically responsive membranes (ERMs) as of 15 October 2020: (**a**) annual publications of ERM papers based on Scopus database, and (**b**) article-type-based distribution (research vs. review). (**c**) Materials used for designing ERMs in previous studies. The list of the research articles was obtained by searching with keywords “(‘electrically conductive’ or ‘electrically responsive’ or ‘electro-conductive’ or ‘electro-active’ or ‘electro-responsive’) and ‘membrane’ and ‘water purification’” in Scopus, then manually removing unrelated articles.

Owing to this enhanced performance and versatility, ERMs recently garnered increasing attention from membrane researchers and is considered one of the most promising solutions to tackle problems in current membrane-based water purification processes [15]. As shown in Figure 1a, a search in the Scopus database gave about 100 research articles to date. The published papers continue to increase over the years since 2012, reflecting a consistently growing interest. In addition, several review papers associated with ERMs have also been published [15,16,29,30,31,32,33] (Figure 1b). Comparatively, nano-enabled ERMs are at an early stage of development. Therefore, we believe that it is time to give an overview of nano-enabled ERMs, focusing on novel 1D- and 2D-nanomaterial-based ERMs, their working principles, fabrication, application, and future perspectives. We hope that this article will help inspire researchers and path them in their research to develop more attractive nano-enabled ERMs.

## 2. Nanomaterials for Electro-Responsive Membranes

ERMs have been conventionally fabricated using various conductive materials, such as conductive polymers, carbon-based nanomaterials, metal, and metal oxide nanomaterials [15,31,34,35]. One- and two-dimensional nanomaterials were recently introduced as new building blocks for designing high-performance next-generation membranes [7,8,9]. Nanomaterials such as CNTs and graphene not only possess excellent properties for water transport, but also high electrical conductivity [8,9]. Therefore, many previous ERM studies have employed these carbonaceous nanomaterials for membrane separation (Figure 1c). In addition to these carbonaceous nanomaterials, more recently, 2D MXenes, molecular sheets with intrinsic good conductivity and superb capacitance made of metal carbides, transition nitrides, or carbonitrides, have also been evaluated for their feasibility as a building block for ERMs [26,36]. Interestingly, MXenes could also be made from different arrangements of transition metals such as molybdenum and titanium.

**Figure 2:**
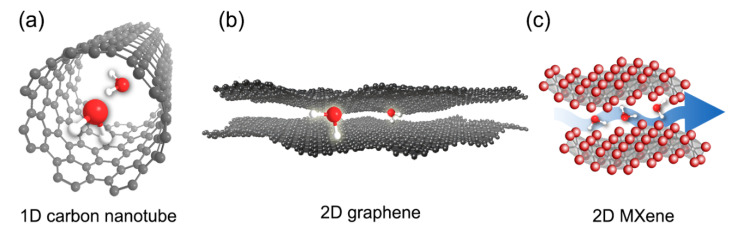
Illustration of 1D and 2D nanomaterials used for electrically responsive membranes: (**a**) carbon nanotube, (**b**) graphene, and (**c**) MXenes, showing water transport through them.

Table 1 displays the property comparison of these three nanomaterials. Morphologically, CNTs are 1D hollow-type nanomaterials made of carbon (Figure 2a). Depending on the number of carbon wall, CNTs can be classified into single-walled CNTs (SWCNTs) and multi-walled CNTs (MWCNTs) [37]. CNTs have a high aspect ratio; the diameter of CNTs ranges only a few nanometers, but the length can amount to millimeters long [38]. The hydrophobic inner channel of CNTs offers an almost frictionless transport pathway for water molecules [9]. Thus, if both tips (ends) of CNTs are opened, ultrafast water transport can theoretically occur through the inner channel of CNTs. Moreover, CNTs have different inner channel diameter ranging from ~1 nm to 50 nm, which can potentially be used to produce membranes with different pore sizes [9,38]. In terms of electrical conductivity, CNTs demonstrate remarkable value ranging from 10^4^–10^5^ S/cm, which is approximately 100 times higher than copper [39]. Other 1D materials that have been used in the preparation of ERMs include Ti_4_O_7_ [40,41,42] and lead zirconate titanate (Pb_(1-x)_Zr_x_TiO_3_ [43].

Unlike CNTs, graphene-based derivatives and MXenes are 2D in structure, with a thickness that can go down to atomic level (Figure 2b,c). Despite the small thickness, they demonstrate remarkable mechanical strengths. Exfoliated graphene-based and MXene nanomaterials exist in nanosheets with lateral sizes that are adjustable from ~100 nm to a few micrometers [8,9], giving these 2D nanomaterials a high aspect ratio. In addition, owing to this high aspect ratio, ultra-thin membranes be fabricated using these 2D nanomaterials. Nanochannel galleries for excellent water transport and molecular sieving can also be formed when these nanosheets are restacked together [8,9]. In particular, similar to CNTs, the hydrophobic nanochannel walls formed using graphene derivatives can provide a low friction pathway for water transport [44]. When it comes to electrical conductivity, pristine graphene and MXene can theoretically reach up to 10^6^ and 10^4^ S/cm, respectively [8,45]. These electrical conductivities are comparable, if not higher, than CNTs. However, due to the top-down approach commonly used for bulk synthesizing, a large number of defective sites is inevitably formed in these 2D nanomaterials, resulting in much lower obtainable electrical conductivity. For example, reduced graphene oxide (rGO) exhibits only a conductivity of 304 S/cm, which is a far cry from that of pristine graphene [46]. Nevertheless, the rich surface chemistry of these materials enables versatile tuning of the electrical conductivity, which renders them compelling for ERM application.

## 3. Preparation of Electro-Responsive Membranes

**Figure 3:**
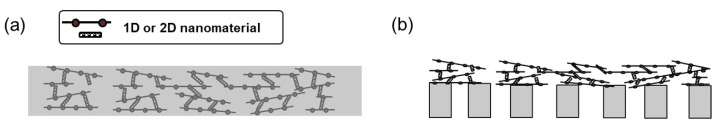
Schematic diagram illustrating the two most commonly adopted membrane structures for incorporating 1D and 2D nanomaterials in electrically responsive membranes. (**a**) polymeric matrix incorporated with nanomaterials, and (**b**) nanomaterial-deposited on porous substrate.

For the preparation of nanomaterial-based ERMs, mainly two strategies have been adapted: First, incorporating nanomaterials into polymer matrices (Figure 3a). Second, depositing the layer of nanomaterials on membrane supports (Figure 3b). The fabrication of ERMs via the first strategy can be generally completed by liquid-phase non-solvent induced phase separation method [29]. The phase inversion method involves (1) dispersing the conductive nanomaterial into a polymer dope solution, (2) casting the polymer dope solution, and (3) inducing phase separation by extracting the solvent from the casted polymer dope solution through a non-solvent induced coagulation [42]. Besides liquid-phase inversion, other various methods, such as interfacial polymerization [47], polymer crosslinking [48], electro-polymerization [49], and vapor phase polymerization etc. [50], have been used to prepare mixed-matrix ERMs.

To obtain the ERMs via the second strategy, several different fabrication methods have also been employed. The most common method to deposit electrically conductive nanomaterial layers on support substrates is vacuum-(or pressure-) assisted filtration method [26,51,52,53]. Prior to conducting vacuum-assisted filtration, a homogeneous nanomaterial dispersion needs to be prepared. For this, a proper solvent should be selected, or the nanomaterials should be functionalized to increase its solubility in a particular solvent [54]. In addition to vacuum- (or pressure-) assisted filtration, there are several alternative methods for nanomaterial deposition. Omi et al. [55] fabricated CNT-based ERMs via a vacuum-assisted layer-by-layer method. Also, electrophoretic deposition [56] and chemical vapor deposition [17] have been used to obtain hollow fiber type rGO-CNT and CNT-based ERMs, respectively.

## 4. Versatility and Working Principle of Electro-Responsive Membranes

**Figure 4:**
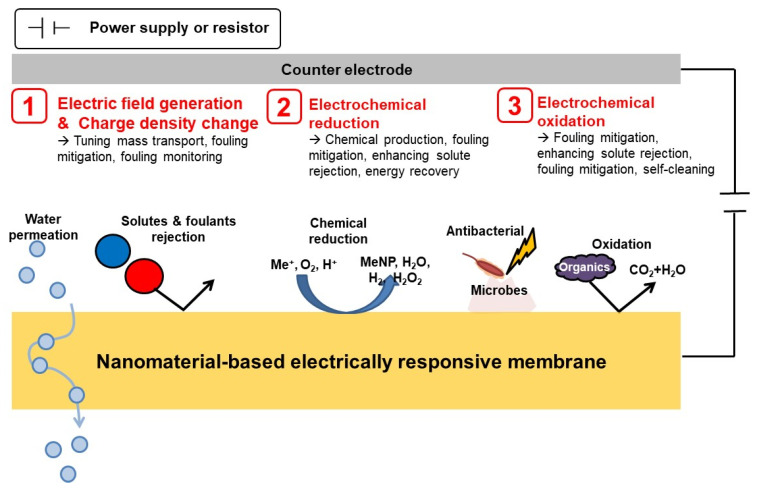
Schematic diagram illustrating the versatile functionalities and working principles of the electrically responsive membranes.

Through leveraging the electrical conductivity of the nanomaterials, ERMs improve their separation performance and become “smarter” by being able to administer designed functionalities. Hence, before we review the separation performance and application of ERMs, we would like to discuss the mechanisms behind ERMs. In this section, brief explanations on versatile functionalities and its related working principles are provided. Figure 4 illustrates some smart abilities and relevant principles of ERMs. To begin with, by regulating the external voltage applied to ERMs, the electric field and charge density on the surface of ERMs can be tuned [24,26]. Consequently, the tendency of water transport, ionic or molecular sieving, surface fouling properties can be altered by varying the strength of electrostatic repulsion and the electrical double layer on the membrane surface [24,26,32].

Next, when external power is applied to the circuit between ERMs and the counter electrodes, some solutes of interest in the aqueous solution can be electrochemically reduced at the surface of the ERMs [22,27,49,57]. For example, through electrochemical reduction, heavy metal ions can be removed from water by precipitating on the surface of ERMs [51]. In addition, hydrogen gas and hydrogen peroxide can be produced by electrochemical reduction on the surface of ERMs [22,57]. If the ERMs are applied as cathodic electrodes of microbial fuel cells, electrical energy can be directly and spontaneously recovered from organic matters via a coupled redox reaction between anodic organic oxidation and cathodic oxygen reduction [27].

Lastly, electrochemical oxidation of contaminants in aqueous solution can occur at the surface of ERMs with the assistance of an externally applied voltage [58,59]. There are two routes for electrochemical oxidation–direct and mediated [60]. In direct oxidation, organic constituents are degraded by radicals, typically hydroxyl radicals, generated at the surface of ERMs. On the other hand, in mediated oxidation, some oxidizing agents, such as chloride and hydrogen peroxide, are required to oxidize organic contaminants. The oxidizing agents can be electrochemically generated in situ or externally added to the system.

## 5. Applications of Electrically Responsive Membranes

Through the above-mentioned working principles, ERMs can control water and solute permeability, create solute rejection enhancement, and enhance fouling mitigation, monitoring and self-cleaning. Below, we would like to provide a discussion on the process made in each application of ERMs.

### 5.1. Fouling Mitigation

**Figure 5:**
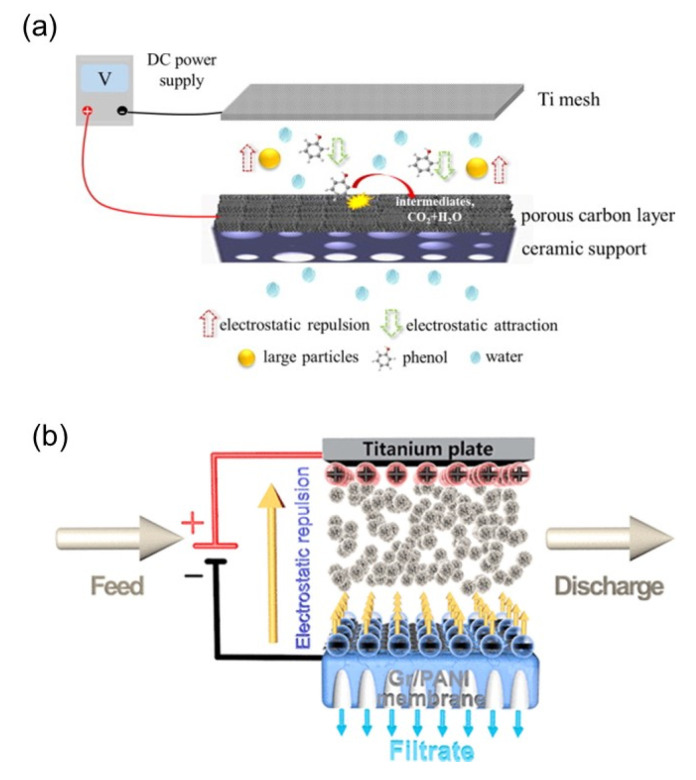
Fouling mitigation by electrically responsive membranes, (**a**) as illustrated by Chen et al. [69] and (**b**) as illustrated by Li et al. [73]. Reproduced with permission from [69,73].

Fouling is an unavoidable phenomenon in every membrane process. This is because particles, contaminants and materials referred to as foulants in the aqueous solution are physically blocked at the membrane surface as water separates out. This leads to a concentration of foulants at the feed side and accumulation on the surface and pores of the membrane. The build-up of foulants on the membrane results in flux decline, poorer selectivity, shortened membrane lifespan, and increased operating costs. Recent efforts to mitigate fouling include intervention by leveraging repulsive interactions between membranes and foulants [61], membrane surface modifications that enhance hydrophilicity [62], electrostatic charges [63], and roughness [63], as well as the use of new membrane materials [64]. One of the promising strategies to mitigate membrane fouling is the use of new membrane materials which are electro-responsive. This is achieved by the addition of conductive materials into the polymer matrix of the membrane, or the synthesis and fabrication of inorganic ERMs. The electrical conductivity of an electro-responsive-based membrane is harnessed for fouling prevention. In this process, electrical potentials are applied to the surface of the membrane to reduce attraction between the foulant and membrane in order to make the membrane less susceptible to fouling. Since most foulants are negatively charged [65], increasing the negative charge of the membrane by an application of electric potential would cause an electrostatic repulsion between many different types of foulants and the charged membrane. Fortunately, most of the 1D and 2D nanomaterials are hydrophilic in nature even without the application of electric current, and thus confer some of their hydrophilic properties on membranes. Hydrophilic membranes have inherent antifouling properties due to their ability to repel hydrophobic foulants, preventing hydrophobic foulants from accumulating on membranes [66,67,68]. Such obstruction and water loving nature of the membrane increase membrane rejection property and water permeation across the membrane. The intrinsic hydrophilic membrane property accorded by 1D and 2D conductive nanomaterials decreases membrane fouling propensity, especially hydrophobic foulants. Consequently, the application of an electric potential to increase surface hydrophilicity would further reduce hydrophobic interactions between the membrane and hydrophobic foulants. An illustration of the working principle of fouling mitigation by ERMs is shown in Figure 5.

For example, Chen et al. [69] fabricated a porous carbon membrane (PCM) by coating porous carbon derived from metal-organic frameworks (MOFs) onto ceramic support. The antifouling property was tested using polystyrene (PS) microspheres as a model for suspended particles. Results showed that membrane fouling by PS microspheres was mitigated when a current of 1.5 V was applied to the membrane, owing to the electrostatic repulsion between the foulants and membrane. This was shown by the comparable antifouling effect and enhanced water flux when the membrane was coated with PS microspheres to create a similar charge effect. Ho et al. [70] also developed a graphene oxide (GO) and MWCNTs (GO/MWCNTs) membrane by blending GO and MWCNTs. The fouling mitigation performance was tested (chemical oxygen demand (COD), phosphorous, color, total suspended solids (TSS), turbidity, and total dissolved solids (TDS)) while passing an electric field continuously or intermittently through the membrane. Various electric field strength was applied in a continuous mode, while a fixed electric field at 5 min interval was applied in an intermittent mode. Although the fabricated membrane showed moderate antifouling properties without the passage of an electric field, a significant improvement in the antifouling property was observed after the electric field was applied, especially in the continuous mode. An increased antifouling effect was observed as the electric field strength increased due to a stronger electrostatic repulsion created between the foulants and membrane. However, this was capped at a threshold electric strength, where subsequent increase did not induce sufficient electrostatic repulsion to provide the further antifouling effect. Du et al. [52] investigated the antifouling capacity of their CNT/nanofiber composite hollow fiber (CNT-HF) membranes synthesized via an electro-assistance method. The antifouling property was tested against both colloids and dissolved organic matters (DOMs). The supply of a 2V negative voltage to the CNT-HFMs improved the flux loss to 6.2% from the initial 28.2% observed when no current was applied after operating for 2 h. However, with the supply of a 2V positive voltage, the water flux declined at a faster rate to 35.3%. This was attributed to the electrostatic repulsion created between the foulants (colloids and DOM) and membrane when a negative voltage was applied. Conversely, the passage of positive voltage neutralized the repulsion force and instead created an attractive force, which enhanced foulant-membrane interaction. Other studies on electric repulsion for fouling mitigation include the work by Sun et al. [21]. In this work, the electrically conducting graphene hydrogel membrane (GHM) was used to mitigate fouling by humic acid (HA) and clay.

Fouling mitigation by ERMs is not limited to only particulate/colloidal, and organic fouling, but also biofouling. Membranes used in many water treatment applications are extremely sensitive to microbial attack, leading to bacterial adhesion and subsequent biofilm formation. The best approach to mitigating biofouling is to proactively prevent microbial attachment on the membrane surface than to passively carry out membrane cleaning after biofouling occurs. In this regard, ERMs have shown to be effective in preventing microbial attachment for controlling biofouling. Previous reports include the work by Ronen et al. [71] who studied bacterial attachment on ERM by changing the applied electrical potentials. The results of their work showed that hydrogen peroxide (H_2_O_2_) was produced at low electrical potential due to oxygen reduction. It was claimed that the H_2_O_2_ production played a crucial role in microbial deactivation by disrupting the membrane structure of the microbial cells, thereby reducing microbial attachment propensity to the membrane surface. de Lannoy et al. [72] also reported a lowered fouling propensity in their fabricated polyamide/MWCNT (PA/MWCNT) nanocomposite membranes when an electric potential of 1.5 V was applied. The disruption of microbial cell growth was linked to the fouling mitigation of the PA/MWCNT membrane.

Recently, Li et al. [73] prepared a conductive graphene/polyaniline (Gr-PANI) membrane with a one-step electrochemical process involving placing the PANI membrane and graphite foil vertically in an electrolyte 1 cm away from both the cathode and anode. The application of current to the electrode stripped Gr from the graphite foil and deposited it on the PANI membrane. The antifouling properties of Gr-PANI were evaluated by filtering yeast solution on the membrane and evaluating the flux decline as a potential difference of 1V was applied. Results showed a 1.4-fold increase in the mean flux as compared to the pristine PANI membrane. The antifouling effect was reported to be due to the creation of electrostatic repulsion between the yeast solutes and the Gr-PANI membrane. Jiang et al. [74] coated CNTs on polytetrafluoroethylene (PTFE) membrane to produce an ERM. The antifouling property of the membrane was investigated in both capacitor and resistor modes by using surface water. Both modes showed that membrane fouling can be effectively mitigated due to direct and indirect oxidation, Coulombic repulsion, and joule heating. A microbial attachment was reported to be prevented by disruption of microbial cell structures (direct oxidation) and production of reactive oxygen species (ROS) (indirect oxidation), which caused cell deactivation before and upon contact with the membrane. Similarly, electrostatic repulsion as a result of Coulombic repulsion can repel microbial cells from the membrane surface. In the resistor mode, joule heating occurred when the temperature was raised above 50 °C. The generated heat generated could result in microbial deactivation and thus prevent microbial attachment on the surface of the membrane.

### 5.2. Self-Cleaning

**Figure 6:**
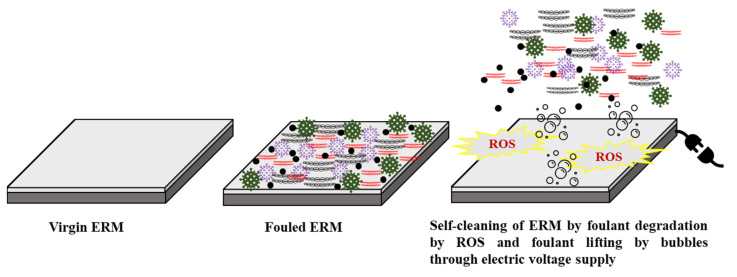
An illustration of the self-cleaning properties of ERMs by applying an electric current.

Membrane fouling cannot be completely prevented but it can be delayed to a certain extent. Sooner or later, any membrane process would require membrane cleaning to recover flux loss to fouling. In most practices, membranes are cleaned after the flux has decreased up to 15% of the original flux. This leads to frequent closure of the water treatment process for clean-in-place practice and maintenance. Cleaning chemicals are also often used and this amounts to a significant portion of the operating costs. The reduction of cleaning chemical use will not only reduce costs incurred for the purchase of cleaning chemicals but also reduce the detrimental impact of discharge of cleaning chemicals on the environment and human well-being. Thus, to reduce the amount of chemicals used in membrane-based water treatment processes, the self-cleaning capability of ERMs have been explored. The self-cleaning function of ERMs is achieved by the production of ROS, which degrades or oxidizes foulants on the membrane surface. The generation of gas bubbles also helps lift foulants off the surface of the membrane. For example, Karkooti et al. [75] used a pressure deposition method to laminate a thin layer of polyaniline (PANI)-rGO on polyethersulfone (PES) and investigated the fouling propensity and self-cleaning ability toward alginate. The application of an electric potential of 9 V to the fouled membrane achieved a flux recovery by 97.47%. They attributed this self-cleaning property to the production of gas bubbles such as oxygen, hydrogen, and even chlorine in the presence of chloride ions in the solution. In addition, the self-cleaning efficiency was accredited to the ROS production such as hydroxyl radical. ROS can react with organic materials on the membrane surface to break them down and dislodge them from the membrane surface. Subtil et al. [76] also prepared PANI-rGO via a phase inversion method. The self-cleaning property was tested against HA and bovine serum alginate (BSA). The prepared PES-PANI(with camphorsulfonic acid)-rGO (0.2 g) membrane showed a self-cleaning efficiency of 81.3 ± 3.6%. The self-cleaning property was again attributed to the production of nanobubbles which attached to the foulants, causing them to dislodge in the presence of electrostatic repulsion as the bubble grew. The self-cleaning performance of a GHM was investigated by Sun et al. [21]. The membrane was fouled with BSA and self-cleaned for 60 min in the presence of Na_2_SO_4_ and an applied voltage of −1.0 V. The flux recovery ratio (FRR) of the GHM was 99.0 ± 1.4%. However, self-cleaning efficiency decreased as foulant concentration increased and cleaning time decreased. ROS production through oxygen reduction and electrical repulsion was also believed to have contributed to the self-cleaning ability. Wang et al. [77] evaluated the self-cleaning efficiency of a hybridized CNT-functionalized ceramic (h-CNT/CN) membrane after the membrane was fouled with alginate. The supply of 3 V voltage for 15 min to the membrane recovered the water flux almost completely. The removal of alginate from the fabricated membrane was also reported to be due to the production of reactive chlorine species which helped degraded the alginate on the membrane. The degradation of the tightly bound alginate led to the loosening of the alginate and its eventual removal. de Lannoy et al. [72] tested the self-cleaning efficiency of the PA-CNT membrane by using *Pseudomonas aeruginosa* as a foulant. The application of an alternating potential of 1.5 V resulted in a self-cleaning efficiency of between 92% and 100%. It was hypothesized that the self-cleaning property was a result of the local pH and electrical double layer instabilities brought by the electro-oxidation processes. Lalia et al. [78] made an electro-conductive carbon nanostructured/PVDF (CNS/PVDF) membrane by a vacuum-assisted filtration method. The stability of the membrane was achieved by thermal treatment, which helped PVDF bound to the CNS. The CNS-PVDF membrane was fouled with yeast suspension, after which it was self-cleaned by applying a 2 V potential in the presence of a 10 g/L NaCl solution with the membrane as the cathode and stainless steel as the anode. The self-cleaning capacity of the membrane was attributed to the formation of microbubbles on the membrane surface. A 98% flux recovery was reported after the first cycle due to the removal of foulants from the membrane by the microbubbles. This finding was superior to another previous report by Hashaikeh et al. [57] who deposited electrically conductive MWCNTs on the PVDF membrane. An illustration of the self-cleaning working principles of ERMs is presented in Figure 6.

### 5.3. Fouling Monitoring

Fouling monitoring is becoming an important aspect of the membrane-based water treatment process as it provides online membrane fouling status, which is vital for early fouling intervention, optimization of feed water pretreatment, and the implementation of cleaning procedures. Early detection of fouling implies that system operators can clean the membrane with water back-washing or mild chemicals as opposed to seasoned fouling which needs to be cleaned by hasher conditions and stronger chemicals. Avoiding excessive cleaning chemicals would increase membrane longevity and reduce chemical use in the water treatment system. Unfortunately, early detection of fouling on the membrane is challenging in high flux membranes [79]. Efforts are usually centered on feed water fouling potential monitoring. Feed water fouling potential monitoring does not always translate to real membrane fouling because of either under- or over-estimation of fouling potential. Thus, direct monitoring of membrane fouling potential appears to be an effective and efficient approach. ERMs show promise in monitoring fouling on membranes in real time by measuring resistance changes on the surface of the membrane through an electrochemical process.

**Figure 7:**
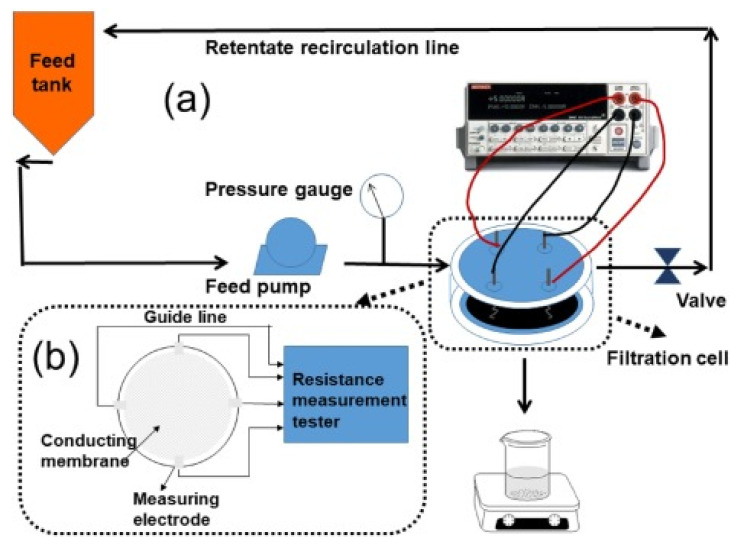
An example of fouling monitoring by electrically responsive membranes. Reproduced with permission from [80].

The feasibility of ERMs to monitor and detect fouling on a water treatment membrane was studied by Yuan et al. [80] (Figure 7). In this work, oxidized MWCNTs (o-MWCNTs) with GO and PVDF membrane was prepared by the phase inversion method. Foulants on o-MWCNTs/GO/PVDF membrane was monitored by measuring membrane sheet resistance. The sheet resistance was reported to decrease as foulants accumulated on the membrane surface. In another study by Zhang et al. [81], electrical impedance spectroscopy (EIS) technique was used to monitor fouling of latex beads on coated single-walled/double-walled CNTs (SW/DWCNT) layer on a PES substrate. Results showed that this technique was quite effective in detecting subtle changes in foulant concentrations on the membrane without notably affecting the membrane flux or selectivity. The onset and progression of accumulation of latex beads on the membrane resulted in an increase in membrane resistance. Compared to the conventional four-point electrode used in the EIS systems, the conductive membrane can be used as a working electrode. Using the conductive membrane as an electrode allows for a clearer differentiation between the electrode and the electrode/liquid interface. In another related work, Ahmed et al. [82] also used the EIS technique to monitor early colloidal silica fouling on a CNS-silica-polyvinyl alcohol (CNS-Si-PVA) membrane. The progression of colloidal fouling on the CNS-Si-PVA membrane was monitored as a function of the increasing membrane capacitance by using the membrane as an electrode to couple to another counter electrode.

### 5.4. Organic Contaminant Removal by Electrochemical Oxidation

**Figure 8:**
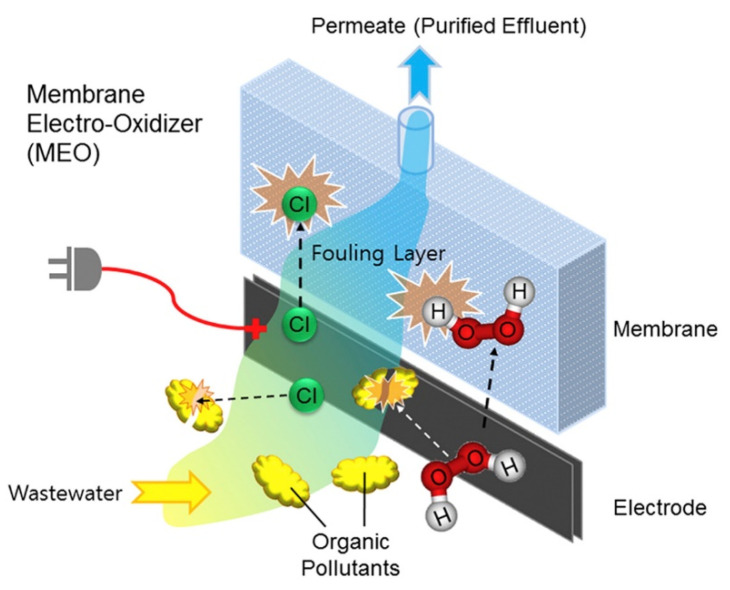
Organic contaminant removal by electrochemical oxidation by electrically responsive membranes. Reproduced with permission from [84].

The additional use of chemicals for the removal of contaminants is a major challenge in the membrane system. In this respect, the application of ERMs with electrochemical oxidation capacity is gaining strong interest recently. This is because electrochemical oxidative ERMs are not only able to mineralize contaminants but also enhance the mass transport of a contaminant-free solution. Contaminant removal is achieved by the production of ROS which are strong oxidants that are capable of degrading a wide range of contaminants on the membrane surface and the ability to induce electron transfer from the contaminant to the membrane to bring about a degradation of the contaminant. Figure 8 is an example of the mechanism of ERM for the removal of organic foulants by produced reactive chlorine species and hydrogen peroxide during electrochemical oxidation. Chen et al. [69] used an electrochemically active PCM to degrade organic pollutants, phenol, and anionic dyes, such as methyl orange (MO). A high phenol removal rate of >85% was achieved when a potential of +2.0 V was applied to the membrane in pure water containing Na_2_SO_4_. Even in the presence of high saline water, phenol removal of around 80% was achievable with a supply of +1.0 V potential. However, a further increase in voltage did not improve phenol removal. For MO removal, the PCM degraded the MO anionic dye by first adsorbing the dye solutes on the PCM and subsequently degraded them with the passage of +1.0 V potential. Duan et al. [49] also tested the electro-oxidation ability of PANI/carboxylated CNTs (PANI/CNT-COOH) membrane for methylene blue (MB) removal. Results showed 90% electrochemical oxidation of MB when a potential of 3 V was applied. Liu et al. [83] prepare conductive graphene nanoplatelets with CNTs in a PTFE (GNP:CNT/PTFE) membrane. They investigated the degradation of three organic contaminants (tetracycline, phenol, and oxalate) and found a correlation between the anodic potential and electro-oxidation kinetics. An increased in anodic potential resulted in a corresponding increase in electro-oxidation kinetics for the three contaminants in the order of phenol > oxalate > tetracycline. However, a significant increase in electro-oxidation kinetic was not observed when the voltage increased to above 0.8 V.

### 5.5. Controlling Water Permeability

Compared to fouling and contaminant removal, fewer studies are focused on demonstrating controllable water and ion permeation across ERMs by adjusting externally applied voltage [73]. In a previous research performed by Zhou et al. [85], the transport of water molecules across the nanochannel with conducting filaments created in a laminar GO membrane can be adjustable by applying external voltage to the GO membrane. The GO membrane used in their study was fabricated by first depositing GO laminates on silver porous support filter electrode, and then attaching gold film electrode on the top of the GO laminates. To fulfill controlled water transport in the prepared GO membranes, conductive filaments were created via tunable electrical breakdown. These filaments played a critical role in controlling water transport in the GO laminates. When an electric current was applied, an electric field generated around the conductive filaments enabled ionization of water molecules inside the GO nanochannels, resulting in regulated water molecule permeation.

**Figure 9:**
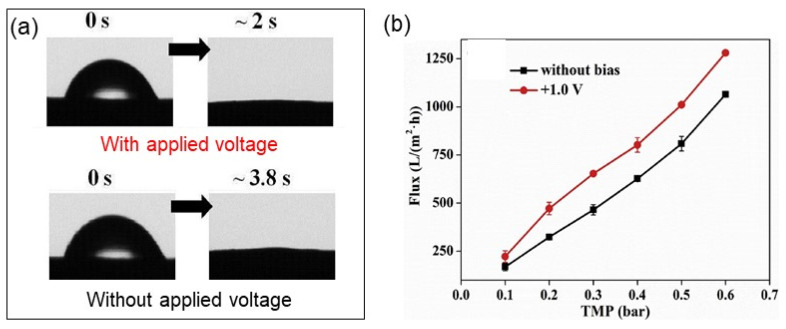
(**a**) Changes in surface water contact angle of carbon nanotube-based hollow fiber membranes (CNT-HFMs) with (**upper**) and without (**bottom**) an applied voltage over time. (**b**) Changes in water flux across the CNT-HFMs with/without an applied voltage as a function of transmembrane pressure. Reproduced with permission from [86].

More recently, a CNT-based hollow fiber ERM was fabricated using surface-functionalized CNTs and polyvinyl butyral polymer via a wet-spinning method [86]. The CNT-based hollow fiber ERM exhibited enhanced water permeability due to improved surface hydrophilicity with the supply of an external voltage of 1.0 V (Figure 9).

### 5.6. Enhancing Ion and Organic Dye Molecule Rejections

**Figure 10:**
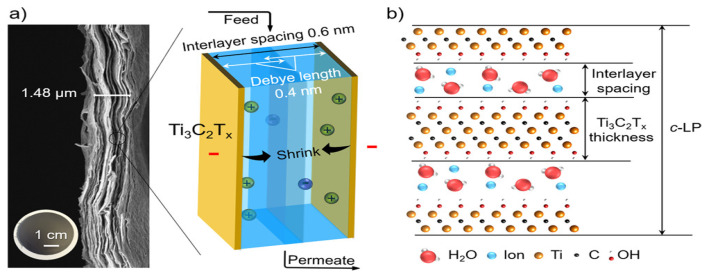
(**a**) Cross-section scanning electron microscopy image of laminar Ti_3_C_2_T_x_ MXene ERM and a diagram illustrating the behavior of ions within the electrical double layers of laminar Ti_3_C_2_T_x_ Mxene ERM when a negative voltage is applied. (**b**) Molecular model showing water molecules and ions within layers of laminar Ti_3_C_2_T_x_ Mxene ERM. Reproduced with permission from [26].

ERMs can be used to improve the ion and organic molecule rejections via various electrochemical mechanisms such as electrostatic repulsion, and electrochemical reduction and oxidation. A previous study conducted by Ren et al. [26] demonstrated for the first time the tunable size of nanochannels formed in a laminar Ti_3_C_2_T_x_ MXene membrane by applying a various external voltage to the MXene membrane. The nanochannels were tightened by a negative applied voltage, whereas a positive voltage caused an enlargement of the nanochannels (Figure 10). This voltage-induced resizable MXene nanochannel enabled an efficient control of ion/molecule permeation across the membrane. This MXene-based ERM achieved an improved MB dye rejection of over 99.6% with an applied voltage of −0.5 V.

In addition, conductive Ni-deposited GO (Ni-GO) membranes were used for the removal of Congo red organic dye. Without an applied voltage, the Ni-GO ERM showed a low Congo red dye rejection efficiency of only 60–80%. However, by applying an external voltage of ~30 V, the Ni-GO ERM accomplished an increased rejection efficiency of over 98%. According to the study, the increased dye rejection efficiency is the consequence of a synergistic effect brought by electrochemical hydrogen gas bubble generation from the deposited Ni layer and the increased electrostatic repulsion driven by the external direct current (DC) power [57].

Also, in another study [51], conductive CNT-incorporated PVDF ultrafiltration membrane on stainless steel mesh support (CNT-PVDF-SS UF) were developed. During the filtration process with an applied voltage of ~5 V, CNT-PVDF-SS UF ERMs were able to efficiently remove toxic chromium metal ions, Cr(VI), by electrostatic repulsion and electrochemical reduction of Cr(VI) to Cr(III) on the membrane surface [42].

### 5.7. Wettability Mitigation in the Membrane Distillation Process

**Figure 11:**
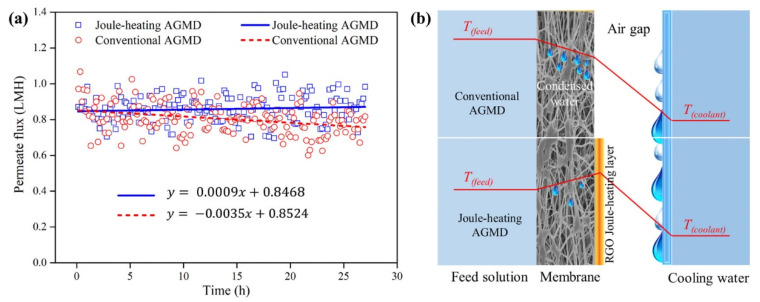
(**a**) Permeate flux of conventional air-gap membrane distillation (AGMD) and an AGMD equipped with rGO Joule-heating membrane in the orientation with rGO layer facing against air-gap side (Joule-heating AGMD). (**b**) The schematic diagram of temperature profiles of the conventional AGMD and Joule-heating AGMD. Reproduced with permission from [28].

Recently, a conductive rGO membrane with PTFE support was employed for membrane distillation (MD) to mitigate membrane wetting [28]. Since the laminar rGO layer possesses a good electrothermal property, it can be self-heated when a DC power is directly supplied to the membrane. As described in Figure 11, by setting up the rGO membrane in the orientation where the rGO Joule-heating layer faced the air-gap compartment for condensation in an electrothermally driven MD process, membrane wetting resulting from capillary condensation was alleviated during the MD operation by applying DC power to the rGO membrane. Although the rGO Joule-heating layer faced against the air-gap side, over 90% of the heat generated was reported to flow from the rGO layer into the feed side to heat up the feed water. As a result, the permeate flux was more stable as compared to a conventional MD system with hydrophobic membranes.

### 5.8. Energy Recovery in A Bioelectrochemical System

ERMs have also been installed as a cathode electrode in bioelectrochemical systems (BESs) to drive simultaneous water purification and energy recovery from organic wastes [87]. In the BESs, organic contaminants in wastewaters can be oxidized by electrochemically active bacteria at an anode electrode, generating electrons and protons. The protons migrate through the electrolyte, while the electrons are transferred through the external circuit connecting the ERM, which is being used as a cathode electrode. The protons and electrons are used for cathodic reduction reaction at the ERM. Through this mechanism, chemical energy contained in wastewater can be recovered by the BES. There are various forms of recovered energy, such as electricity, hydrogen peroxide, and hydrogen gas depending on the cathodic reduction reaction at the ERM.

**Figure 12:**
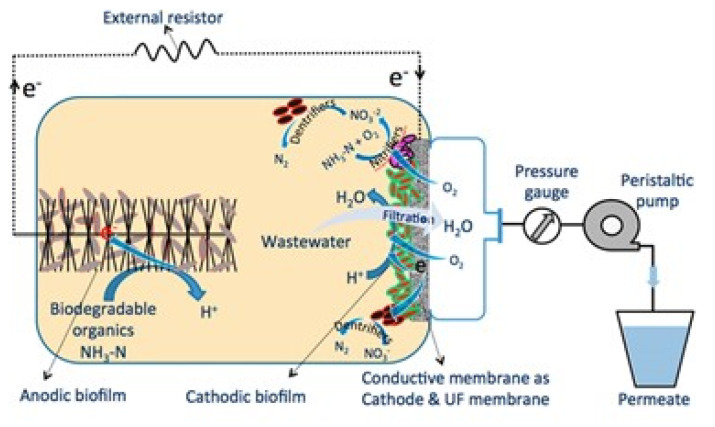
Schematic of bioelectrochemical system equipped with a multi-walled carbon nanotube-based ultrafiltration membrane as cathode electrode for filtering wastewater and oxygen reduction reaction. Reproduced with permission from [27].

MWCNT- and rGO-based ERMs were developed as cathodic filter electrodes for the BESs (Figure 12). Malaeb et al. [27] fabricated MWCNT-based UF ERM and employed it in a BES for electricity generation. The BES equipped with the MWCNT-based ERM generated a maximum power density of 0.38 W/m^2^ as well as efficiently producing high-quality permeate (97% COD removal, 97% NH_3_-N removal, and 91% total bacteria removal) from domestic wastewater.

In addition, Huang et al. [88] set up an rGO-incorporated PVDF microfiltration (MF) ERM as a cathode electrode in a BES. At the rGO-based ERM, NOx- and O_2_ reduction took place, resulting in electricity generation and denitrification. The BES with rGO-based ERM achieved a maximum power density of 0.35 W/m^2^ with a removal efficiency of about 97% for COD and about 95% for total nitrogen content.

## 6. Current Challenges and Outlook

Leveraging nanomaterial-based ERMs currently seems to be a promising strategy to achieve high-efficient and more sustainable water purification processes. Although the nanomaterial-based ERMs is still at an early stage of development, the results demonstrated in lab-scale studies are quite interesting and encouraging. However, in order to step up and attain practical application of nanomaterial-based ERMs, future research needs to focus on solving several critical issues, such as stability, safety, scalability, and economic feasibility. Although the operating conditions vary based on different membrane processes, many membrane processes are operated under relatively harsh conditions with applied pressure and crossflow velocity. As such, having good mechanical stability for nanomaterial-based ERMs is vital. Currently, the stability of nanomaterial-polymer composite ERMs may not be an issue due to the cross-linking of nanomaterials with the polymer matrix. However, the mechanical stability of ERMs with conductive nanomaterials as the active layer is of concern. Current research on nanomaterial-based ERMs is conducted on a lab-scale with limited information on mechanical stability, especially nanomaterial coated/deposited ERMs. It is important that research interest in ERMs should not be limited to performance alone, but mechanical stability in practical applications.

Furthermore, concerns about the use of nanomaterials in daily life remain given that their long-term impacts on human health and the environment are not extensively studied [89]. Thus, another challenge to overcome for the commercialization of nanomaterials-based ERMs would be to ascertain the safety of nanomaterials and to set up appropriate guidelines for use and disposal. Particular attention should be given to the potential bioaccumulation of these nanomaterials. Due to the time duration required for this type of study, computer simulation experiments could be conducted to predict the ecotoxicity profile of nanomaterials used in ERMs, both at the manufacturing sites and point-of-use.

Scalability is another factor impeding the practical application of nanomaterial-based ERMs. For ERMs which incorporate conducting nanomaterials into polymer matrices, material dispersibility is a major issue as nanomaterials are prone to aggregate within the polymer matrix, affecting the electrical conductivity uniformity and the overall performance of the ERMs. Also, conducting nanomaterials can exhibit interfacial compatibility issues with organic polymers. Therefore, a thorough assessment is required to match the conducting nanomaterials to the right polymer matrix. In a situation where conducting nanomaterials are used as an active layer on a substrate, it is challenging to fabricate large-scale ERMs without defects. This is because water permeation through a membrane is usually inversely proportional to the membrane thickness. Therefore, manufacturing thin layers of nanomaterials as active layers can lead to the generation of defects on the membrane. The larger the membrane is fabricated, the higher the chance of producing more defects. Thus, research efforts should focus on the preparation of large area defect-free ERMs.

Along with the above-mentioned challenges, the economic viability of ERMs is another critical hurdle that needs to be overcome to realize the potential of nanomaterial-based ERMs. Because polymeric membranes are well-established, the cost of polymeric ERM and commercial membranes will generally be cheaper than nanomaterial-based ERMs. At present, the market price of nanomaterials is high. However, with increasing interest in nanomaterial-based applications, it is expected that the price of nanomaterials would drop, making ERMs more cost-competitive in the future. Similarly, the electrical resistance of nanomaterial-based ERMs is dependent on the conductivity and thickness of the membrane. This causes nanomaterial-based ERMs to experience a rapid potential drop. To maintain sufficient charge density across the membrane surface, a higher potential is required [15], but this will further increase the energy requirement of the ERMs. Nevertheless, a thorough economic assessment of nanomaterial-based ERMs is necessary to elucidate the long-term advantages of the membrane, taking into account their lower propensity to fouling, self-cleaning ability, higher water production, and lower frequency of membrane replacement etc. Perhaps the long-term benefits of nanomaterial-based ERMs would overweigh its capital cost and energy consumption.

Finally, most ERMs are used as an electrode in combination with a counter electrode in an electrochemical cell. Therefore, a new membrane module design is needed to accommodate the counter electrode used in ERM application. Current module designs do not have the appropriate space to accommodate the counter electrode.

## 7. Conclusions

This perspective paper provides an overview of the recent advances in the field of ERMs and discusses the challenges to be addressed in order to realize more practical ERMs. Since the first demonstration of the ERM concept, ERMs have become a promising strategy for resolving some critical issues that conventional membrane-based water purification processes are facing. Owing to their smart and versatile abilities, ERMs see many exciting applications for fouling reduction, tuning mass transport, self-cleaning, and high-efficiency organic contaminant oxidation. The rise of nanomaterials has helped accelerate the advancement in nano-enabled ERMs. Electrically conductive 1D and 2D nanomaterials have pushed the boundary of ERMs, affording new membrane designs and elevating their performance to offer new opportunities beyond that of conventional polymeric membranes. However, stronger efforts are still required to resolve challenges to do with stability, scalability, and costs. We hope that by summarizing the current state-of-the-art membrane designs and providing brief insights into the difficulties that lie ahead, we are able to inspire new nanomaterials and open new opportunities to enhance the performance of ERMs as well as seeing the technology through commercialization.

## Figures and Tables

**Table 1:** Properties of conductive 1D and 2D nanomaterials [8,39,45,46].

Nanomaterial	Structure	Electric Conductivity (S cm^−1^)	Young’s Modulus (TPa)
CNT	1D carbon	10^4^–10^5^	0.93–1
Graphene	2D carbon	10^6^	1
rGO	2D carbon	30^4^	0.25
MXenes	2D Ti_3_C_2_T_x_	About 10^4^	0.33
